# Supplementary material for: A stochastic framework of neurogenesis underlies the assembly of neocortical cytoarchitecture
Source: eLife. 2019 Nov 18;8:e51381. doi: 10.7554/eLife.51381 (PMC6968929; doi:10.7554/eLife.51381)
Supplement: Transparent reporting form [file elife-51381-transrepform.docx]

***eLife’s* transparent reporting form**

We encourage authors to provide detailed information *within their submission* to facilitate the interpretation and replication of experiments. Authors can upload supporting documentation to indicate the use of appropriate reporting guidelines for health-related research (see [EQUATOR Network](http://www.equator-network.org/%20)), life science research (see the [BioSharing Information Resource](https://biosharing.org/)), or the [ARRIVE guidelines](http://www.plosbiology.org/article/info:doi/10.1371/journal.pbio.1000412) for reporting work involving animal research. Where applicable, authors should refer to any relevant reporting standards documents in this form.

If you have any questions, please consult our Journal Policies and/or contact us: [editorial@elifesciences.org](mailto:editorial@elifesciences.org).

**Sample-size estimation**

- You should state whether an appropriate sample size was computed when the study was being designed
- You should state the statistical method of sample size computation and any required assumptions
- If no explicit power analysis was used, you should describe how you decided what sample (replicate) size (number) to use

Please outline where this information can be found within the submission (e.g., sections or figure legends), or explain why this information doesn’t apply to your submission:

Our experimental data provides a detailed description of the relative frequencies of diverse pyramidal cell lineage configurations in the developing murine cortex. Given the descriptive nature of the dataset, *a priori* power calculations where not possible, since the existence and frequency of such diversity of configurations is one of the central findings of our study, and thus, prior knowledge could not be used to estimate the sample size required for their faithful description. For these reasons we used a large sample size that reached a stable quantification of the relative fractions of most lineage configurations observed, so that the addition of further replications did not significantly change our central observations.

**Replicates**

- You should report how often each experiment was performed
- You should include a definition of biological versus technical replication
- The data obtained should be provided and sufficient information should be provided to indicate the number of independent biological and/or technical replicates
- If you encountered any outliers, you should describe how these were handled
- Criteria for exclusion/inclusion of data should be clearly stated
- High-throughput sequence data should be uploaded before submission, with a private link for reviewers provided (these are available from both GEO and ArrayExpress)

Please outline where this information can be found within the submission (e.g., sections or figure legends), or explain why this information doesn’t apply to your submission:

A detailed description of the number of brains and lineages quantified in each experiment is listed in Table S1, which can be found at the end of the main document file. Criteria for data exclusion are clearly stated in the main text when applicable. The clonal nature of our datasets implicates a great variability in the total number of lineages obtained in each brain, consequently returning high variability in the relative fractions of the different configurations observed. For that reason, we present our descriptive data as fractions of total lineages observed across all brains used.

**Statistical reporting**

- Statistical analysis methods should be described and justified
- Raw data should be presented in figures whenever informative to do so (typically when N per group is less than 10)
- For each experiment, you should identify the statistical tests used, exact values of N, definitions of center, methods of multiple test correction, and dispersion and precision measures (e.g., mean, median, SD, SEM, confidence intervals; and, for the major substantive results, a measure of effect size (e.g., Pearson's r, Cohen's d)
- Report exact p-values wherever possible alongside the summary statistics and 95% confidence intervals. These should be reported for all key questions and not only when the p-value is less than 0.05.

Please outline where this information can be found within the submission (e.g., sections or figure legends), or explain why this information doesn’t apply to your submission:

All statistical tests used, exact p-values, dispersion and precision measures, and number of samples (n) are reported in Table S1, which can be found at the end of the main document file.

(For large datasets, or papers with a very large number of statistical tests, you may upload a single table file with tests, Ns, etc., with reference to sections in the manuscript.)

**Group allocation**

- Indicate how samples were allocated into experimental groups (in the case of clinical studies, please specify allocation to treatment method); if randomization was used, please also state if restricted randomization was applied
- Indicate if masking was used during group allocation, data collection and/or data analysis

Please outline where this information can be found within the submission (e.g., sections or figure legends), or explain why this information doesn’t apply to your submission:

All samples of each individual experiment were treated as a single common group and therefore this information is not applicable to our submission.

**Additional data files (“source data”)**

- We encourage you to upload relevant additional data files, such as numerical data that are represented as a graph in a figure, or as a summary table
- Where provided, these should be in the most useful format, and they can be uploaded as “Source data” files linked to a main figure or table
- Include model definition files including the full list of parameters used
- Include code used for data analysis (e.g., R, MatLab)
- Avoid stating that data files are “available upon request”

Please indicate the figures or tables for which source data files have been provided:

The exact numerical values of all datapoints presented in the figures are listed in Table S1, which can be found at the end of the main document file. All parameters used in our mathematical modeling are described in the main text and/or the Methods section.
